# Intermittent Fasting—A Healthy Dietary Pattern for Diabetic Nephropathy

**DOI:** 10.3390/nu14193995

**Published:** 2022-09-26

**Authors:** Ming Yang, Wei Chen, Liyu He, Di Liu, Li Zhao, Xi Wang

**Affiliations:** 1 Department of Nutrition, Xiangya Hospital, Central South University, Changsha 410008, China; 2 Department of Nephrology, The Second Xiangya Hospital of Central South University, Changsha 410011, China; 3 Department of Reproduction and Genetics, The First Affiliated Hospital of Kunming Medical University, Kunming 650032, China; 4 National Clinical Research Center for Geriatric Disorders, Xiangya Hospital, Central South University, Changsha 410008, China

**Keywords:** intermittent fasting, diabetic nephropathy, mitochondria, ketone body, autophagy

## Abstract

Diabetic nephropathy (DN), a metabolic disease, is characterized by severe systemic metabolic disorders. A unique dietary pattern, such as intermittent fasting (IF) has shown promising protective effects on various metabolic diseases, such as diabetes and cardiovascular and nervous system diseases. However, its role in regulating kidney disease, especially in DN, is still being investigated. Here, we summarize the current research progress, highlighting the relationship between IF and the risk factors for the progression of DN, and discuss the potential mechanisms by which IF improves renal injury in DN. Finally, we propose IF as a potential strategy to prevent and delay DN progression. Abbreviation: DN: Diabetic nephropathy; IF: Intermittent fasting; CPT1A: Carnitine palmitoyltransferase 1A; L-FABP: Liver-type fatty acid-binding protein; STZ: Streptozotocin; LDL: Low-density lipoproteins; HIIT: High-intensity interval training; CKD: Chronic kidney disease; ACEI: Angiotensin-converting enzyme inhibitors; ARB: Angiotensin receptor blockers; MDA: Malondialdehyde; mtDNA: Mitochondrial DNA; UCP3: Uncoupling protein-3; MAM: Mitochondria-associated endoplasmic reticulum membrane; PBMCs: Peripheral blood mononuclear cells; ERK1/2: Extracellular signal-regulated kinase 1/2; DRP1: Dynamin-related protein 1; β-HB: β-Hydroxybutyrate; AcAc: Acetoacetate; GEO: Gene Expression Omnibus; NCBI: National Center for Biotechnology Information; mTORC1: Mechanistic target of rapamycin complex 1; HMGCS2: 3-Hydroxy-3-methylglutaryl-CoA synthase 2; GSK3β: Glycogen synthase kinase 3β; AKI: Acute kidney injury; CMA: Chaperone-mediated autophagy; FGF21: Fibroblast growth factor 21.

## 1. Introduction

Timely food consumption is the most essential requirement for the survival and reproduction of all animals. Over time, the gradual acquisition of physiological and behavioral adaptations by animals, such as hibernation, has ensured their survival during food scarcity. The liver and adipose tissue are the essential organs of energy storage and metabolism and adjust the metabolic state of the whole body according to eating patterns [1,2,3]. The role of intermittent fasting (IF) in delaying disease progression has recently attracted increasing attention from researchers. Studies have shown that IF improves the body’s overall health, enhancing disease resistance [4,5,6,7].

**Figure 1:**
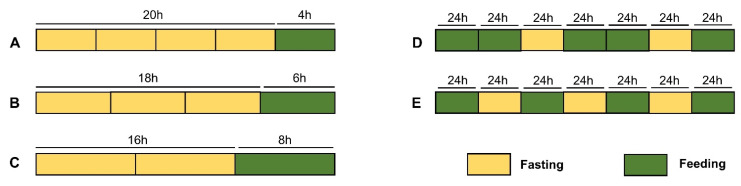
**Patterns of intermittent fasting.** (**A**–**C**) Time-restricted eating: 16 h fast followed by an 8 h eating period (**A**); 18 h fast followed by a 6 h eating period (**B**); 20 h fast followed by a 4 h eating period (**C**). (**D**,**E**) Alternate 24 h fasting with 24 h eating: eating for 5 days of the week and fasting for 2 days (**D**); eating for 4 days of the week and fasting for 3 days (**E**).

IF is less restrictive and easier to follow in daily life diet than conventional caloric restriction. The two predominant patterns of IF include: (1) time-restricted feeding, which contains three variants—16/8, 18/6, and 20/4—16/8 consists of a 16 h fast followed by an 8 h eating period, 18/6 consists of fasting for 18 h followed by 6 h eating, and 20/4 consists of fasting for 20 h followed by 4 h eating [8]; and (2) alternating 24 h fasting with 24 h feeding, and the 5/2 or 4/3 systems, in which one eats for 5 days of the week and fasts for 2 days, or eats for 4 days and fasts for 3 days [8] (Figure 1). Recently, IF has also been shown to modulate systemic metabolic status through multiple mechanisms, thereby delaying the progression of various diseases, such as cardiovascular [9,10], neurological [11,12], and autoimmune [13,14,15] diseases. The role of IF in diabetic nephropathy (DN), a metabolic disorder, is now being investigated in depth. In this review, we summarize the current research progress on the importance of IF in the alteration of DN progression, which includes lipid metabolism disorders and hypertension, and further discuss the molecular mechanism underlying IF-mediated effects on DN progression. Finally, we also hypothesize the possibility that IF is a therapeutic means to delay the progression of DN.

## 2. Methods

This is a narrative review. We focused on the following keywords that were searched in PubMed: diabetic nephropathy (DN), diabetes, kidney, intermittent fasting (IF), hypertension, lipid, mitochondria, ketone body, and autophagy. However, we acknowledge that such narrative reviews cannot rule out selection bias.

## 3. IF and Risk Factors for Progression of DN

### 3.1. IF and Lipid Metabolic Disorder

Lipid metabolism disorders often manifest in patients with DN, marked by increased serum lipid levels and ectopic lipid deposition in the kidney [16,17,18,19]. In addition, lipid metabolism disorders are shown to aggravate diabetic kidney injury by causing lipid deposition and increased lipid droplet volume in renal tissues of patients with diabetic nephropathy diagnosed by biopsy. This effect is accompanied by the significantly decreased expression of carnitine palmitoyltransferase 1A (CPT1A), acyl-CoA oxidase, and liver-type fatty acid-binding protein (L-FABP) compared to the control [16]. Our previous studies have also shown increased lipid deposition in renal tubular cells in streptozotocin (STZ)-induced diabetic mice or db/db mice, as well as an accompanying increase in serum lipids compared with control mice or in HK-2 cells treated with a high concentration of glucose [18,20]. In contrast, the pharmacological inhibition of renal lipid deposition effectively delays the progression of DN and kidney injury [21]. Therefore, maintaining lipid metabolism homeostasis and inhibiting lipid deposition is a potential novel therapeutic strategy for preventing and treating DN.

Dietary control is a crucial factor for maintaining lipid homeostasis. Glucose exhaustion during fasting leads to the utilization of ketones produced by fatty acid conversion for energy, which is the phenomenon that primarily supplies energy to the cells [22,23]. During fasting, the depletion of glycogen stores in the liver activates gluconeogenesis, leading to decreased insulin levels, and increased glucagon secretion, thereby promoting the lipolysis of triacylglycerols in adipocytes [22,23]. These processes encourage the consumption of lipids, leading to weight loss and changes in lipid metabolism. Multiple experiments have also shown how IF improves whole-body lipid metabolism and reduces body weight. Wilson et al. have demonstrated that IF significantly reduced weight gain, lipid deposition, and downregulated serum low-density lipoprotein (LDL) levels compared with the control or high-intensity interval training (HIIT) groups [24]. A similar result was observed when participants following alternate day fasting showed a marked decrease in fat content and serum triglyceride concentration at week 12 compared with the control group. This was accompanied by increased serum adiponectin and decreased leptin levels [25]. In addition, fasting during Ramadan significantly reduced body weight, blood glucose, and triglyceride levels and reduced serum levels of inflammatory factors (IL-2 and TNF-α) [26]. These pieces evidence suggest the effectiveness of IF in improving lipid metabolism and lowering lipid levels, which are key factors delaying the progression of DN.

### 3.2. IF and Hypertension

Hypertension is also a common clinical manifestation of chronic kidney diseases and exacerbates kidney injury and DN progression. The prevalence of hypertension in diabetic patients is twice as high as that in average people and gradually increases with chronic kidney disease (CKD) progression. Hypertension occurs in up to 90% of end-stage renal disease (ESRD) patients [27]. Studies have shown that the progression of hypertension in DN is correlated to increased proteinuria [28], while nocturnal hypertension occurs preferentially over proteinuria [29,30]. In a randomized, double-blind study of 1513 patients, losartan was evaluated in type 2 DN patients, and it was shown that losartan reduced the doubling of serum creatinine and significantly reduced proteinuria [31]. Angiotensin-converting enzyme inhibitors (ACEI) and angiotensin receptor blockers (ARB) have become the priority treatment for patients with early DN without contraindications.

The accumulated clinical and basic research evidence also suggests that IF plays a crucial role in controlling blood pressure. Erdem et al. have shown how IF significantly lowers office and ambulatory blood pressure values compared with controls [32]. A similar study highlighted how, in hypertensive and control groups, fasting decreases arterial pulse pressure by 17.2% and 9.3%, increases serum glutathione levels by 56.8% and 52.6%, and decreases malondialdehyde (MDA) levels by 24.3% and 25.7%, respectively [33]. Moreover, a 5-week, randomized, crossover, isocaloric, true calorie-controlled feeding trial also showed that early time-restricted feeding could reduce morning systolic and diastolic blood pressures by 11 ± 4 mm Hg and 10 ± 4 mm Hg, respectively [34]. Interestingly, this blood pressure reduction was similar to some blood pressure-lowering drugs. In the animal model, the impact of IF on blood pressure was evident, as rats subjected to IF displayed significant reductions in heart rate and blood pressure within one month, which subsequently remained low, along with increased plasma corticotropin and corticosterone levels compared to the control [35]. This observation suggested that IF induced stress responses that caused a systemic alteration in metabolic status through the endocrine system. Moreover, IF has also been shown to improve right ventricular systolic and diastolic functions (their abnormalities are significant pathophysiological changes of pulmonary hypertension). Prisco et al. performed a metabolomics study and showed that IF alters the gut microbial composition, significantly increasing the level of *Lactobacillus*, and reduces right ventricular lipid accumulation, which enhances the right ventricular function [36]. These pieces of evidence suggest that IF plays a vital role in maintaining blood pressure, which, in turn, can effectively delay the progression of DN.

## 4. Molecular Mechanism of IF Alleviation of DN Progression

### 4.1. IF and Mitochondria

The kidney, a hypermetabolic organ, contains abundant mitochondria to provide energy to maintain the hypermetabolic state [37,38]. Multiple studies have confirmed the existence of abnormal mitochondrial morphology and tubular cell functions in DN [39,40,41,42,43]. The mitochondrial dysfunction of renal tubular cells in a diabetic state leads to decreased cellular ATP synthesis [44], increased oxidative stress [45,46], and the activation of inflammation [47,48], thereby aggravating the DN-associated kidney injury. Conversely, maintaining the stability of mitochondrial function effectively delays the progression of DN. Therefore, maintaining mitochondrial function stability is also a potential therapeutic strategy for treating DN. To date, several studies have shown how IF maintains mitochondrial homeostasis to prevent or delay the progression of diseases. Weir et al. have revealed that IF prolongs lifespan by promoting mitochondrial network remodeling, which subsequently promotes fatty acid β-oxidation in the mitochondria [49]. Moreover, it has also been shown that during fasting, the expression of CPT1 (a key enzyme in mitochondrial fatty acid β-oxidation) was considerably increased [50,51]. In addition to fatty acid β-oxidation, the abnormal release of mitochondrial DNA (mtDNA) into the cytoplasm also aggravates renal inflammatory injury in DN. Interestingly, IF upregulates mtDNA content [52] and reduces the level of 8-oxodG (a marker of mtDNA oxidative damage) [53]. In addition, IF can also regulate mitochondrial function through different pathways.

The 24-hour fasting process significantly increases the mRNA expression of uncoupling protein-3 (UCP3, a mitochondrial inner membrane protein that is involved in energy dissipation) in muscle compared with the control group [54]. The mitochondria-associated endoplasmic reticulum membrane (MAM) is a newly discovered sub-organelle structure, composed of mitochondria, the adjacent ER, and proteins [55,56]. Sepúlveda et al. have demonstrated the disruption of MAM integrity during the metabolic transition from fasting to feeding, which induces mitochondrial fission and reduces mitochondrial crest density in human peripheral blood mononuclear cells (PBMCs) [57]. In addition, the response to IF in different tissues is varied. In the rat liver, mitochondrial respiratory capacity increases upon IF, accompanied by increased protein carbonyl levels, which protects the heart from oxidative damage, but aggravates oxidative damage in the brain [58]. This difference between tissues may be due to their different energy requirements. This evidence suggests that IF maintains mitochondrial homeostasis by regulating various aspects of mitochondria. Several studies have also shown the renoprotective role of IF in kidney injury involves the maintenance of mitochondrial homeostasis.

In a rat model of acute kidney injury, preoperative fasting maintains the stability of mitochondrial morphology and function, thereby reducing and preventing tubulointerstitial fibrosis [59]. In addition, Morales et al. have shown that compared with the control group, TRF intervention one week before the operation significantly ameliorates renal function in a rat renal ischemia–reperfusion injury model, inhibits the extracellular signal-regulated kinase 1/2 (ERK1/2) signaling pathway, and reduces the progression of tubulointerstitial fibrosis [60]. These TRF-mediated renoprotective effects are associated with mitochondrial oxidative stress inhibition, and reduced DRP1-mediated mitochondrial fragmentation and the activation of the mitochondrial unfolded protein response (UPR) [60]. Although little is known about the IF regulation of mitochondrial function in DN, the relationship between IF and mitochondria and the association between mitochondria and DN highlights these as promising research directions.

### 4.2. IF and Ketone Bodoes

Ketone bodies, including d-β-hydroxybutyrate (β-HB), acetoacetate (AcAc), and 3-carboacetone, are produced primarily through the fatty acid oxidation pathway [61]. The liver has a specific set of enzymes for synthesizing ketone bodies, making the liver the primary site of ketone body production. However, the liver lacks enzymes to utilize ketone bodies [62,63,64]. As an energy product, ketone bodies are mainly utilized by the brain, heart, kidney, and skeletal muscle [65]. Analyzing ten datasets from the Gene Expression Omnibus (GEO) database of the National Center for Biotechnology Information (NCBI) revealed that ketone body metabolism ranked the highest in the KEGG enrichment analysis of DN [66]. Moreover, Li et al. found that diabetic patients with high or normal serum ketone levels had a reduced risk of DN compared with those with low serum circulating ketone levels, which serves as an indicator of kidney injury in diabetic patients [67]. A similar result was also observed in the DN mice model. Tomita et al. demonstrated that in ApoE-knockout mice fed a high-fat diet (a DN model), excessive activation of the mechanistic target of rapamycin complex 1 (mTORC1) leads to a shift in energy metabolism from lipolysis to ketosis in renal tubular cells [68]. Moreover, the treatment of DN mice with empagliflozin (which probably upregulates the endogenous ketone body levels) or a precursor of ketone bodies (1, 3-butanediol) significantly increased renal ATP levels, relieving renal injury. However, the depletion of 3-hydroxy-3-methylglutaryl-CoA synthase 2 (HMGCS2), a rate-limiting enzyme in ketogenesis, partially prevented these protective effects [68]. These observations indicate that the supply of ketone bodies in the kidneys of DN patients is insufficient, and increasing their concentration effectively alleviates diabetic kidney injury. In addition to maintaining renal energy supply, ketone bodies delay renal injury through various pathways. β-HB could target the ATP-binding pocket of glycogen synthase kinase 3β (GSK3β), thereby inhibiting it from exerting its diabetic nephroprotective effect [69]. Our previous study also demonstrated how β-HB supplementation ameliorates cisplatin-induced acute kidney injury (AKI) by inhibiting NLRP3 inflammasome activation and the cGAS-STING pathway [70]. These studies demonstrate how ketone bodies effectively protect against renal damage in DN. IF, as a means of dietary control, significantly affects plasma ketone body content, establishing a direct link between IF and DN.

As mentioned earlier, glucose exhaustion during fasting induces the body to switch from fatty acids to ketones for energy [23]. Toledo et al. have shown that fasting decreases the blood glucose levels to the low normal range and increases ketone body levels [71]. Moreover, metabolomics revealed that the ketone body content in the plasma of subjects undergoing regular IF was significantly higher than that of controls [72]. In addition, the ketone body level was also increased in mice undergoing IF, while a liver-specific knockout of tuberous sclerosis 1 (an mTORC1 inhibitor) resulted in significant defects in hepatic ketone body production and ketogenic gene expression [73]. These results collectively indicate how mTORC1 regulates the ketogenic response during fasting, suggesting ketogenesis is another potentially critical mechanism of IF-mediated DN regulation.

### 4.3. IF and Autophagy

Autophagy leads to the encapsulation of excess or damaged proteins or organelles in the cytoplasm into vesicles that fuse with lysosomes to form autolysosomes, which degrade their encapsulated contents [74,75]. This process is beneficial for cells to renew their organelles and prevent further impairment by damaged organelles. Based on the degraded contents, there are three types of autophagy: macroautophagy [76], microautophagy [77], and chaperone-mediated autophagy (CMA) [78]. Macroautophagy is further divided into mitophagy [37], ER-phagy [79], lipophagy [80], and so on. To date, starvation is the most critical inducer of autophagy. During energy scarcity, cells degrade excess organelles to maintain their energy supply [81,82].

When cells are exposed to high glucose, hypoxia, oxidative stress, and other stimuli, the intracellular autophagy machinery will be activated to remove damaged organelles or proteins, which is essential for cell survival [83]. Although the DN-associated mechanism of autophagy remains to be studied further, many studies have shown that autophagy activity is abnormal in DN. Moreover, autophagy was decreased in renal proximal tubules of STZ-induced early diabetic rats [84]. Similarly, our previous study also showed the inhibition of autophagy in kidney biopsies from DN patients, db/db mice [18], and HK-2 cells treated with high glucose [85], while the activation of autophagy effectively alleviates diabetic kidney injury [86]. This observation indicates that abnormal autophagy is involved in the occurrence of DN.

**Figure 2:**
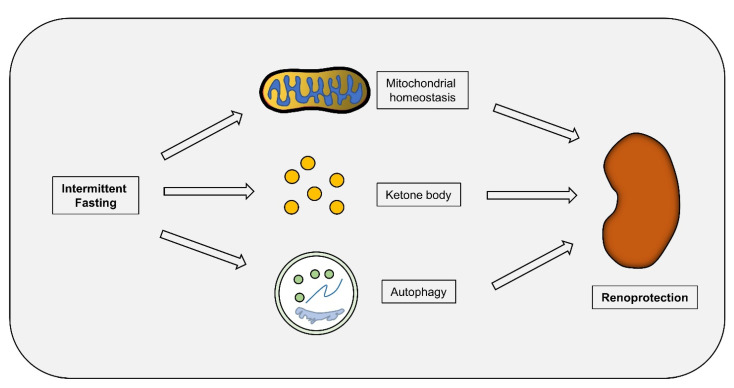
**The potential mechanisms underlying the protective effect of IF in DN progression.** IF dietary patterns maintain mitochondrial homeostasis, increase the production of ketone bodies, and promote autophagy, thereby delaying DN progression.

The relationship between IF and autophagy has been elucidated by the observation that IF could effectively upregulate the activity of autophagy to maintain tissue homeostasis. In a 4-day study involving 11 overweight adults, time-restricted feeding increased the expression of autophagy-associated gene LC3A in the blood [87]. In addition, Byun et al. have shown that fasting-induced expression of fibroblast growth factor 21 (FGF21) in the liver promotes the phosphorylation of JMJD3 at Thr1044 by PKA, which induces the nuclear translocation of JMJD3 and enhances its interaction with nuclear receptor PPARα, thereby increasing autophagy [88]. Moreover, increased β cell death and aberrant autophagic flux were also seen in islets of obesity-induced diabetic mice, and IF restored autophagic flux, improved glucose tolerance, and promoted β cell survival [89]. Moreover, IF also improved the peripheral and central changes induced by a high-fat diet by maintaining the balance of autophagy [90]. The direct evidence linking IF, autophagy, and DN was demonstrated by Gouda et al. in an STZ-induced DN mice model, where apoptosis was increased, and autophagy was inhibited, while fasting restored renal autophagy, reduced apoptosis, and alleviated kidney injury [91] (Figure 2). Although the molecular mechanism underlying autophagy activation by IF in DN remains to be studied further, it is confirmed that IF-mediated autophagy activation is an effective protective strategy against DN.

In addition to controlling the potential mechanisms mentioned above, IF also plays a vital role in regulating biological rhythms [92,93], inflammation [94,95], and oxidative stress [96], abnormalities of which are closely related to DN progression [97]. We believe that further advancements in deciphering the role of IF will gradually reveal the underlying mechanism governing the protective effect of IF in DN.

## 5. Conclusions

DN is a systemic metabolic disorder that can aggravate diabetic kidney injury, while IF can effectively restore metabolic homeostasis and ameliorate the progression of the disease. Here, we summarize the current progress into research into early diabetic rats research progress on the relationship between IF and the risk factors for DN progression. Moreover, we discuss the potential mechanisms by which IF ameliorates renal injury in DN. Finally, we hypothesize that IF may be a potential therapeutic strategy for preventing and delaying DN progression. Despite IF being an essential beneficial lifestyle choice for DN patients, many issues still need further exploration. What molecular players fuel the protective effects of IF on DN? Which mode of IF is most beneficial for delaying the progression of DN? In addition, serious risk factors must be considered in managing the complex association of insulin and fasting, as patients receiving high insulin doses are at greater risk for hypoglycemia during IF. Therefore, accurately regulating the amount of insulin used during fasting is critical for the prevention of hypoglycemia [98]. Despite IF being well studied in various metabolic diseases, its association with DN still needs to be extensively investigated. We hypothesize that the progress of IF research will help establish it as a novel strategy for the prevention and treatment of DN in the future.

## Data Availability

Not applicable.
